# SAL3D: a model for saliency prediction in 3D meshes

**DOI:** 10.1007/s00371-023-03206-0

**Published:** 2024-01-04

**Authors:** Daniel Martin, Andres Fandos, Belen Masia, Ana Serrano

**Affiliations:** Universidad de Zaragoza, I3A, Zaragoza, Spain

**Keywords:** Saliency, Eye tracking, Attention, 3D meshes

## Abstract

Advances in virtual and augmented reality have increased the demand for immersive and engaging 3D experiences. To create such experiences, it is crucial to understand visual attention in 3D environments, which is typically modeled by means of saliency maps. While attention in 2D images and traditional media has been widely studied, there is still much to explore in 3D settings. In this work, we propose a deep learning-based model for predicting saliency when viewing 3D objects, which is a first step toward understanding and predicting attention in 3D environments. Previous approaches rely solely on low-level geometric cues or unnatural conditions, however, our model is trained on a dataset of real viewing data that we have manually captured, which indeed reflects actual human viewing behavior. Our approach outperforms existing state-of-the-art methods and closely approximates the ground-truth data. Our results demonstrate the effectiveness of our approach in predicting attention in 3D objects, which can pave the way for creating more immersive and engaging 3D experiences.

## Introduction

**Fig. 1:**
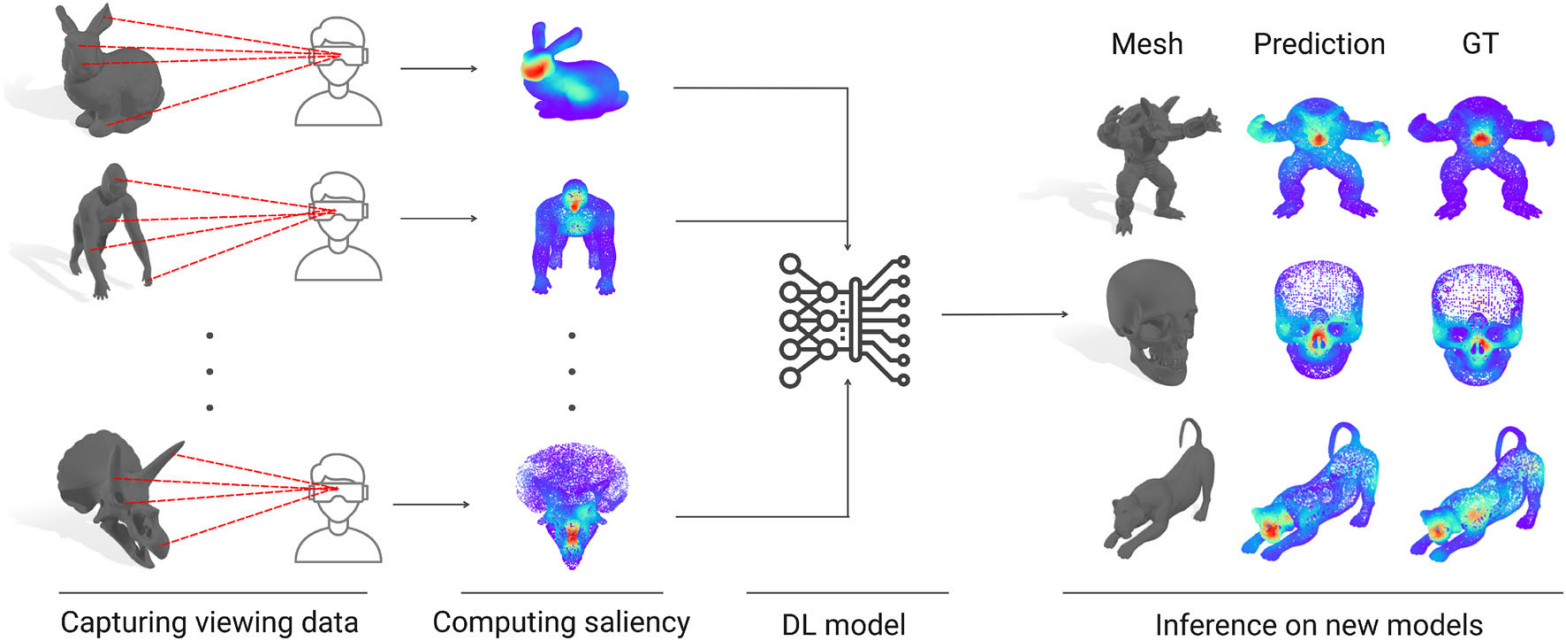
In this work, we present a novel 3D mesh saliency predictor trained on real, captured viewing data. We have captured a large dataset of 32 participants viewing 58 different meshes in a virtual reality-based environment, and have then trained a deep learning-based model to predict saliency in any unseen 3D mesh. We have evaluated our model and compared it to available previous approaches, with our model yielding results that outperform the state of the art

We live in a three-dimensional (3D) world, and the importance of such three dimensions is rooted in our biological evolution: We are designed to comprehend, interact, and process information based on what we perceive in terms of depth, height, and width. Indeed, in our everyday life, we rely on this spatial awareness to enhance our understanding and navigate the world that surrounds us. Common activities like driving a car require processing 3D information to perceive speed and distance to maneuver as required, and many artistic experiences such as painting or video games become significantly more realistic when depicting three-dimensional scenes, as they are closer to real life than their 2D counterpart. Relevance of 3D scenarios is further increased by the recent surge of virtual and augmented reality (VR / AR, respectively), which provide users with the ability to interact with scenes in a manner closer to that of the real world, and often require faithful representations of 3D environments. Our visual system is wired to be directed toward certain elements or features in a scene, both from a bottom-up (e.g., colors, contrast, lines) and from a top-down (e.g., task- or context-dependent) perspective. Therefore, understanding human attention is important to create appealing 3D experiences, e.g., for VR / AR, as well as to foster other applications such as foveated rendering or mesh simplifications, which could alleviate computational costs.

A vast body of literature has resorted to *saliency* to measure attention, as a topological measure of the conspicuity of the different elements of a scene, i.e., the parts that were more likely to draw attention [1, 2]. While many efforts have been done in this regard for 2D content (e.g., conventional images [3–5] or $360^{\circ }$ content [6–8]), much remains to be explored in 3D stimuli. Besides, 3D environments provide many cues that are not present on 2D, like motion parallax or vergence movements [9], and thus what is known from traditional media may not apply to 3D.

Several attempts have been made to analyze visual attention in 3D shapes. However, most of these attempts rely on hand-crafted operators and geometric cues, such as Gaussian curvatures, or global and local rarity, to determine which parts of a 3D mesh would attract more attention [10–13]. These approaches usually succeed in identifying the most conspicuous parts of the geometry, but still suffer from limited expressive capabilities, as they do not model the semantic and top-down cues that play a fundamental role in human viewing behavior. On the other hand, some other works have resorted to data-driven techniques [14–16] to create computational models of attention. Nevertheless, these works have been trained on datasets gathered in rather constrained conditions, far from natural viewing: In some cases, users were required to view static, 3D-printed figures without any possible movement [17] (i.e., using a chin-rest and within fixed distances and viewpoints), while in other cases, users had to view 2D displays that showed a limited number of viewpoints of the meshes [18]. While these works have shown the potential of data-driven techniques for attention modeling, none of them were trained on data that accurately captures natural, human viewing behavior.

To address these limitations, we have first collected what, to our knowledge, is the largest dataset of real gaze data on 3D shapes. It comprises 58 different meshes from several open-source databases, and we have gathered gaze and head data from 32 participants viewing these stimuli for over 20 s each in a VR setting. In contrast to previous works, which often recorded viewing data under highly constrained laboratory conditions (e.g., using chin-rests, or fixing the viewpoint of the shapes), VR allows for easy and efficient manipulation of the stimuli and facilitates gaze data collection, while also offering more natural viewing conditions, including depth perception, motion parallax, or stereo viewing, among others. Leveraging this dataset, we have developed a deep learning-based model built upon a state-of-the-art classification network for pointclouds [19] to predict *saliency* on 3D meshes, which represents the likelihood of viewers directing their attention to different regions of the meshes. An overview of this work can be seen in Fig. 1. We have evaluated the performance of our proposed model with commonly used metrics, and comparing it with existing methods for predicting attention in 3D meshes. Our results show that our model achieves higher accuracy than existing methods.

Our contributions can be summarized as follows:

- We have collected the largest dataset of real gaze data to date, which comprises gaze data from 32 participants viewing 58 different 3D meshes in a VR setup.
- We have built a deep learning-based saliency prediction model upon a state-of-the-art classification network.
- We have qualitatively and quantitatively evaluated our model, which yields more accurate results than previous approaches.

We will make our model and data publicly available to foster future research.

The rest of the manuscript is structured as follows: Sect. 2 provides an overview of the state of the art in predicting and modeling attention. Section 3 is devoted to the capture and processing of our dataset of viewing behavior in 3D meshes. Then, Sect. 4 delves into our proposed saliency prediction model, which is then thoroughly evaluated in Sect. 5. Finally, Sect. 6 summarizes the work and proposes lines for future work.

## Related work

In this section, we first summarize the state of the art in visual attention prediction in both traditional and $360^{\circ }$ images, and then move to existing approaches that address attention prediction in 3D shapes, our main objective.

### Predicting and modeling attention in 2D content

In the last decades, attention prediction has been an active research area. In the late 90’s, Koch and Ullman [2] and Itti et al. [1] introduced their seminal works on *saliency* prediction. They extracted and leveraged low-level cues, such as color, intensity and orientation, to define the most interesting regions of a scene. Since them, several works followed such heuristic-based approach [20, 21]; however, their handcrafted methods have fallen short to effectively mimic human viewing behavior. With the proliferation of data-driven strategies and deep learning techniques, more sophisticated models have arisen [22–27], achieving strikingly better results. Most of them have resorted to the so-called convolutional neural networks (CNN), which allow to encounter and model inherent spatial patterns and features from the stimuli themselves. Lately, *scanpath* (i.e., trajectory of gaze points) prediction has posed as a more sophisticated approach toward attention prediction, where not only the spatial properties of the stimuli are taken into account, but also the temporal evolution of such attention [3–5, 7, 28]. Further, and with the recent proliferation of virtual reality, understanding human behaviors in virtual environments has gained increased attention [29]. Many works have applied the knowledge acquired in traditional content (as aforementioned) to understand viewing behavior in VR, including saliency prediction in $360^{\circ }$ still images [30], saliency prediction in $360^{\circ }$ videos [8, 31], or scanpath prediction [6, 7, 32].

While the stimuli these works have worked with differs from ours, they have proven the potential of data-driven and deep learning approaches toward achieving unprecedented results on attention modeling.

### Predicting and modeling attention in 3D content

While the previous section shows the increasing interest in attention prediction for 2D content, a much narrower body of literature has been devoted to attention modeling in 3D content, despite its importance toward more realistic experiences. So far, most of the existing approaches have been based on statistical methods: Lee et al. [10] defined mesh saliency based on the differences of Gaussian-weighted mean curvatures at different scales. Leifman et al. [11] proposed an algorithm that detected regions that are distinct both locally and globally, while also providing descriptive presentation of the shape. Later, Song et al. [12] introduced a model based on the log-Laplacian spectrum of the mesh, capturing saliency in the frequency domain, while also following a multi-scale approach. Tasse et al. [13] proposed a cluster-based approach to point set saliency detection, by evaluating cluster uniqueness and spatial distribution of each cluster. All of these works build upon the premise that the most conspicuous parts of a shape (i.e., the parts that stand out more) are more likely to draw attention. In a similar fashion, Wu et al. [33] captured geometric features of several regions, and computed local contrast and global rarity (i.e., contrast between features) to obtain mesh saliency. Hu et al. [34] also took into account rarity to obtain a set of salient regions globally distinct from each other. Additionally, mesh saliency can be computed utilizing curvature entropy [35] or curvature co-occurrence histograms [36]. However, all the aforementioned works depend on extracting handcrafted descriptors [10, 37, 38]. Such operations suffer of reduced expressive capabilities, since they only work on geometric space, and do not take into account the context of semantic information of the meshes themselves. Different to them, we address this problem from a data-driven approach: We do not resort to hand-crafted features, but instead train a deep learning-based model to learn from real user data.

Some works have already implemented convolutional neural networks to predict saliency on 3D meshes [14–16], usually based on the features extracted by neural networks designed and used for classification problems. These works have been trained either on a weakly supervised manner, or on datasets either obtained from more low-level geometric properties, or where participants were asked to manually select the interesting regions of the meshes [39, 40], instead of using real gaze information. Indeed, some of these meshes have also been used in other works [17, 18, 41] to study human viewing behavior when looking at different meshes under different conditions (e.g., material, point of view, room lighting). In our work, we aim to model real viewing behaviors, and thus we conduct an eye-tracking guided experiment to capture a dataset of real viewing behavior on 3D meshes to train our model.

## A dataset of viewing behavior in 3D shapes

We aim at understanding viewing behavior in 3D shapes. Previous approaches have either gathered gaze data from 2D pictures depicting different perspectives of 3D models [18], or have used real, physical objects in rather constrained conditions [17]. Virtual reality poses itself as a better suited alternative, since it provides more natural interactions (e.g., stereo viewing and motion parallax) and simpler tools for designing and presenting 3D stimuli to viewers, allows for easier manipulations (e.g., rotating or moving) of such stimuli, and eases capturing attentional behaviors. Thus, we have resorted to VR to develop an experiment to collect head and gaze data from multiple viewers observing a larger set of 3D shapes than previous approaches.

**Fig. 2:**
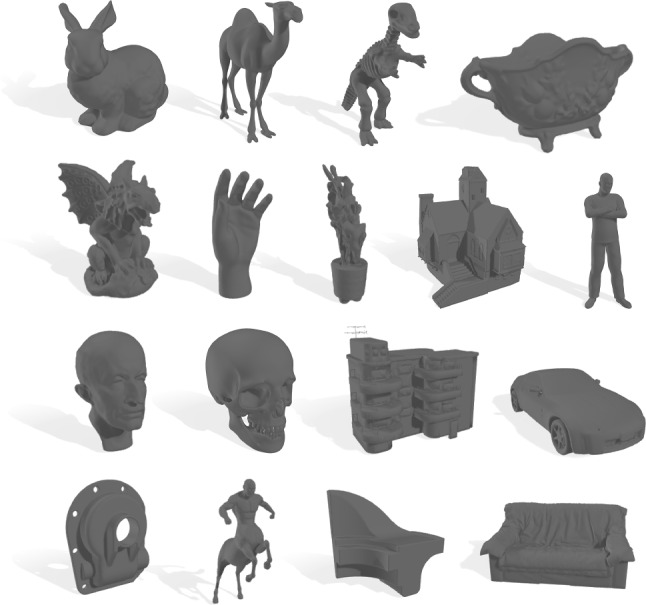
Subset of the 3D meshes used during our experiment. We have collected several meshes from different open-source datasets (see Sect. 3.1), and have conducted a large experiment to gather gaze and head data from 32 different participants viewing those meshes

### Stimuli

We have gathered a total of 58 different 3D meshes from different public databases (Aim@Shape^1^, TOSCA^2^, SHREC 2007^3^, Georgia Tech Models Archive^4^, FREE3D^5^, TurboSquid^6^, CGTrader^7^). They depict humans, animals and creatures, familiar objects, or mechanical parts, among others (see Fig. 2), and are all textureless. Once gathered, we have processed all of them to have the same size and orientation, following the work from Qi et al. [19]. In particular, we have centered and normalized all meshes to occupy a 2-by-2 meter cube. When presented to the participants, all shapes were uniformly colored in a neutral gray color with a light blue background, and with two identical light sources located above the users and slightly to the left and right, respectively [18].

**Fig. 3:**
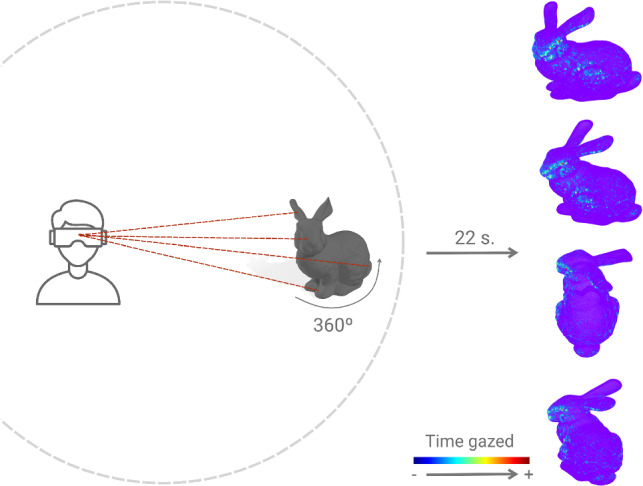
Overview of our experimental setup. Each participant viewed each of the meshes for 22 s, while the mesh was rotating 360 degrees to ensure all parts of the mesh were actually disclosed. Each of such visualizations was transformed into a *gaze map*. Color encodes vertex-wise gaze time

### Participants

A total of 32 participants took part in the experiment. Twenty-three of them identified as male, nine of them identified as female, and none of them identified as non-binary, not listed, or preferring not to disclose their gender, aged between 21 and 56. They voluntarily took part in the study and provided written consent. The participants were naïve to the final purpose of the experiment, and had normal or corrected-to-normal vision. Thirteen participants reported playing video games regularly, and 22 had used a virtual reality headset before. Our data collection procedure was approved by our local Ethics and Research Committee.

**Fig. 4:**
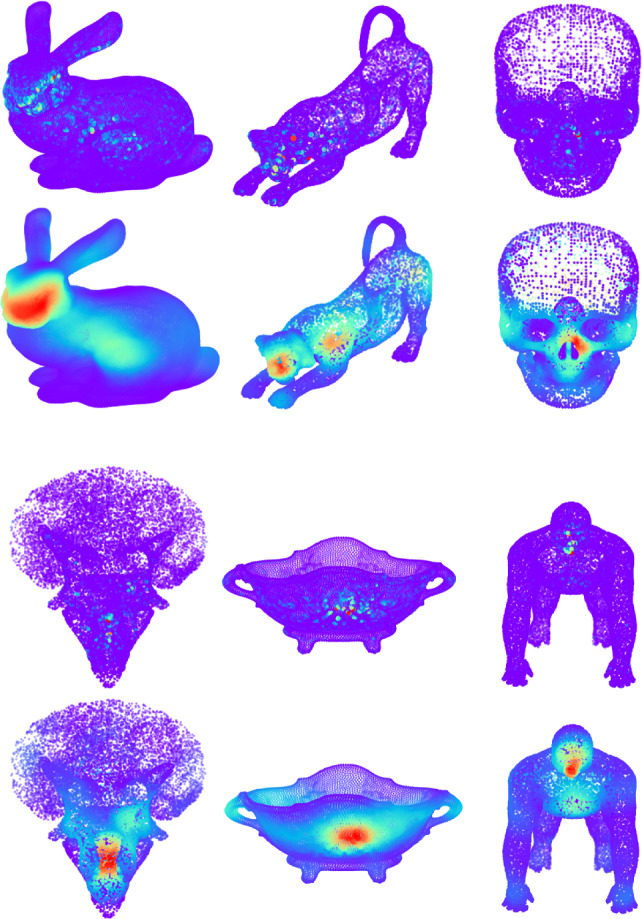
Our computed *gaze maps* (first and third row) are generally sparse, as in traditional media [29]. Therefore, and inspired by previous works, we apply a distance-based mean filter. Second and fourth row shows the result of applying this technique to their sparse counterpart. See Sect. 3.5 for additional details

### Hardware

Our stimuli were presented on an HTC Vive Pro head-mounted display with a nominal field of view of $110^{\circ }$, a resolution of 1440 $\times$ 1600 pixels per eye (2880 $\times$ 1600 pixels combined), and a frame rate of 90 frames per second. We installed in our headset a Pupil Labs^8^ eye tracker to gather gaze information throughout the experiment. Our experimental setup contained two HTC Vive stations to additionally track participants’ head position during the experiment. Our whole procedure was designed using Unity.

### Procedure

We divided our experiment in two different sessions. In the first session, participants were introduced the experiment, gave written consent, and filled a demographic and a pre-experiment sickness questionnaire. Then, they seated on a non-rotating chair, properly adjusted the HMD, and conducted an eye-tracking calibration process. When the calibration was completed successfully, the main experiment began.

In each session, a total of 30 meshes were presented to the participant in a randomized order. To ensure the participant uniformly saw the whole mesh, each mesh was rotated $360^{\circ }$ with respect to its vertical axis. Such rotation was set to last 22 s, which we empirically found to be a reasonable trade-off between the time taken to visualize the mesh and maintaining participants’ engaged (see Fig. 3 for an overview). Note that previous works were limited to showing participants different two-dimensional viewpoints of the same mesh, thus not all parts were uniformly seen. In our case, the rotation allowed for all the viewpoints of the mesh to be equally seen.

We calculated the point of the mesh where participants’ gaze was falling and logged it in real time, along with additional head and gaze information. After the presentation of each mesh, the eye-tracking calibration process repeated, to ensure the gaze information was still being properly collected, and the next mesh was shown. Once all the session’s meshes had been presented, participants had to complete a post-session sickness questionnaire, to assess whether any symptoms appeared through the experiment. The second session of the experiment was identical to the first one, but with the remaining meshes. We asked the participants to take a break of at least 30 minutes between both sessions, to avoid fatigue symptoms which may bias the gathered data.

**Fig. 5:**
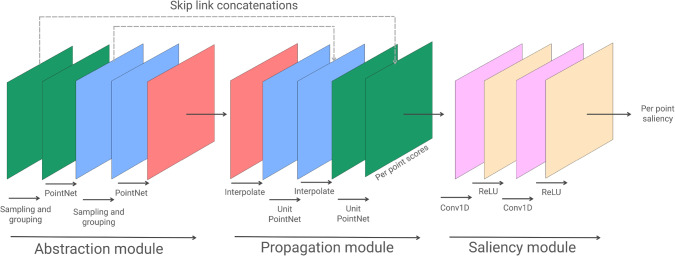
Overview of our model. We build it upon a PointNet++ backbone [19]: We first include three abstraction sets that enable encoding latent features from our meshes. Then, we leverage three propagation modules to propagate such features to the whole original mesh. We finally include a saliency module that allows our network to transform the segmentation features from PointNet++ into saliency. Further details on our model and training procedure can be found in Sect. 4, while we also refer the reader to the original work of PointNet++ [19] for exhaustive information on both abstraction and propagation sets. This figure is adapted from Fig. 2 of the PointNet++ original paper; please refer to Table 1 for further details on the parameters used in the PointNet++ backbone

### Data processing

We logged participants’ gaze and head direction during the whole experiment. We created a *gaze map* per mesh and participant. A total of 1,856 (32 participants $\times$ 58 meshes) gaze maps were obtained. Each of those maps stored how much time the participant was looking at each mesh vertex. To compute such maps, we checked at each timestamp whether the current gaze direction intersected the mesh. If so, we added the elapsed time since the last gaze point to the gazed vertex. We finally aggregated all gaze maps per mesh, and normalized them.

This process nevertheless yielded sparse maps, with many points having received very few gaze points. Thus, and inspired by traditional approaches for smoothing saliency prediction [29] and by gaze density maps [17], we apply a distance-based mean filter. In our case, for each vertex *v* with a gaze time higher than a threshold $\tau = 0.1$, we spread its value to its *N* closest neighbors, proportionally to their distance to *v*. After several experiments, we empirically set $N = 500$, which are the $2.5\%$ vertex’s closest neighbors. We devised this procedure to resemble error ellipsoids around fixations [17]. Figure 4 shows some sample meshes before (first and third row) and after (second and fourth row) applying this smoothing procedure.

## A model for 3D mesh saliency prediction

We have built a 3D mesh saliency prediction model upon a backbone based on the state-of-the-art network PointNet++ [19], since it has shown promising performance in extracting and leveraging inherent point cloud features in tasks, such as classification and segmentation. It partitions a point cloud into local regions, extracts local features from the mesh’s fine geometric structures from small neighborhoods, and then groups those features into larger units to produce higher-level features. This process is repeated several times until enough features are extracted. PointNet++ allows for simultaneously classifying the whole mesh and segmenting its parts. In this work, we resort to the part-segmentation branch of their network. In this section, we briefly describe the PointNet++ backbone (Sect. 4.1) and our final architecture (Sect. 4.2). Then, we go through our loss function (Sect. 4.3) and additional training details (Sect. 4.4).

### PointNet++ backbone

Since PointNet++ is aimed at segmentation and classification of point clouds, its ultimate goal is to learn *set functions* *f* that take sets of points as the input and produce information of semantic interest. The network is composed by a number of *set abstraction* levels and a set of *feature propagation* levels. *Set abstraction* levels process sets of points, abstracting feature vectors from them and producing a new set with fewer elements. *Feature propagation* levels propagate point features obtained from the *set abstraction* levels.

Each *set abstraction* level is made of three layers: a sampling layer, a grouping layer, and a PointNet layer. The *sampling layer* selects a subset of points from the input points by using the Farthest Point Sampling algorithm, and which define the centroids of the local regions. The *grouping layer* builds sets of local regions by finding neighbor points around the centroids defined in the sampling layer by means of the Ball Query algorithm, a method that finds all points that are within a radius from the query point. Finally, the *pointNet layer* encodes local region patterns into feature vectors. In *feature propagation* levels, point features are hierarchically propagated to the original neighbors of the aforementioned subsets, by means of a distance-based interpolation.

**Table 1:** Overview of the main parameters used in the PointNet++ [19] backbone used in our model

		*M*	*r*	*K*	in_channels	*mlp*	group_all
Abstraction module	Set abstraction	512	0.2	64	input_size	[64, 64, 128]	False
	Set abstraction	128	0.4	64	128 + 3	[128, 128, 256]	False
	Set abstraction	–	–	–	256 + 3	[256, 512, 1024]	True
		–	–	–	in_channels	mlp	bn
Propagation module	Feature propagation	–	–	–	1024 + 256	[256, 256]	True
	Feature propagation	–	–	–	256 + 128	[256, 128]	True
	Feature propagation	–	–	–	28 + 6	[128, 128, 128]	True

We refer the reader to the original work for further details on the meaning of these parameters

Please refer to Table 1 for an overview of the parameters used in our backbone, and to the original work [19] for additional details on the architecture and further explanations on the parameters of the original network.

### Model architecture

Our model is composed of three different elements. The first element is a set of three consecutive set abstraction levels from PointNet++, which encode the main features of our meshes. Then, we include a symmetric set of three consecutive propagation levels, which propagate such features to the original point cloud. We finally include a small saliency module, which translates the segmentation features into saliency. An overview of our model can be found in Fig. 5.

### Loss function

Many previous approaches for saliency prediction have resorted to different loss functions or metrics tailored to the specific problem of attention prediction in 2D media, including dynamic time warping (DTW) (e.g., [6]), Pearson’s Correlation Coefficient (CC), Normalized Scanpath Saliency (NSS), or Kullback–Leibler Divergence (KLDiv) (e.g., [8]). Since the latter has shown good performance in different saliency prediction models, we resort to it to optimize our network to learn the spatial distribution of saliency across the mesh. We define our loss function as:

1 $$\begin{aligned} {\text {KLDiv}}\left( G, P\right) =\sum _{x,y,z}\; G_{x,y,z} \;\log \left( \epsilon +\frac{G_{x,y,z}}{\epsilon +P_{x,y,z}}\right) \end{aligned}$$

where $P_{x,y,z}$, $G_{x,y,z}$, are the saliency values of vertex (*x*, *y*, *z*) in the predicted and ground-truth gaze maps, respectively, and $\epsilon$ is a regularization term penalizing zero-valued predictions.

### Training details

**Fig. 6:**
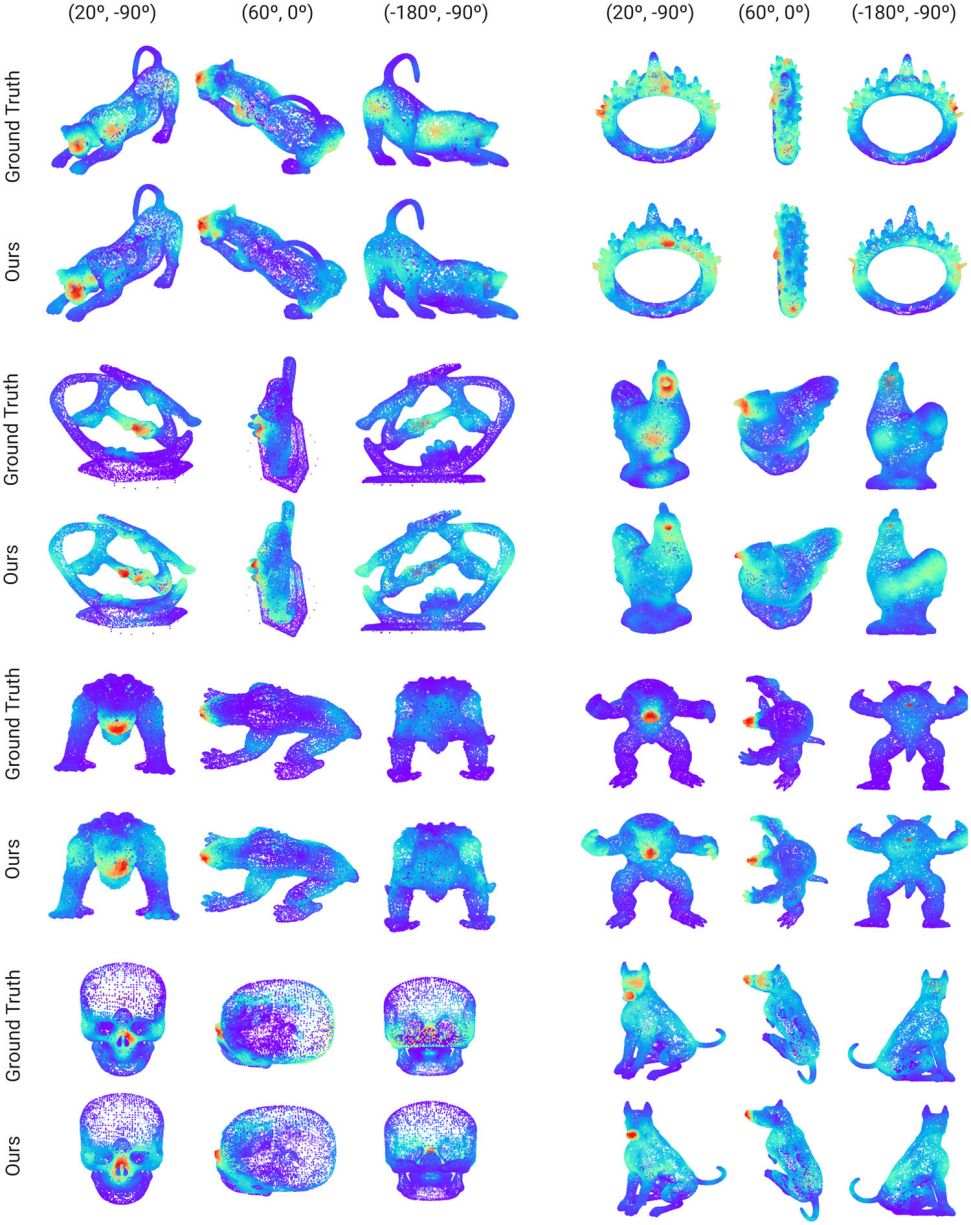
We show here the results from our model in our test set. For each mesh, we show three different viewpoints (parameterized as (elevation, azimuth)) for the ground truth (top rows) and our model’s result (bottom rows). As in previous figures, color codes saliency. Note that our model is able to focus on the most relevant (red) parts, while mostly ignoring the irrelevant (blue) parts. Quantitative evaluations can be found in Table 2, while further discussion can be found in Sects. 5.2 and 6

To train the model, we have first normalized all our meshes—and their corresponding gaze maps—to have 20,000 vertices, which was the mode in terms of mesh size in our dataset. We have trained our model on a NVIDIA RTX 2080 Ti with 11GB of VRAM. We trained our model for 150 epochs, for a total time of approximately and hour and a half, until convergence. We set batch size to 1, and resorted to the Adam optimizer [42], with a learning rate $lr = 10^{-3}$ and a weight decay [43] $w_d = 5^{-4}$. We added the learning rate scheduler StepLR to our optimizer, decaying the learning rate by a factor of $\gamma = 0.8$ every 30 epochs. We have trained our model on 50 (90%) of the gathered meshes, while leaving the rest for evaluation purposes. The 8 meshes corresponding to the test set are displayed in Fig. 6.

## Evaluation

In this section, we perform an exhaustive evaluation of our model. We first briefly review the set of metrics we resort to for our evaluation (Sect. 5.1). Then, we discuss the main results from our model and compare them to some available state-of-the-art works (Sect. 5.2).

### Metrics

To validate our model performance, and compare it to other approaches, we have resorted to three meaningful, well-known metrics commonly used in saliency evaluation, namely Pearson’s correlation coefficient (CC), mean squared error (MSE), and Kullback–Leibler Divergence (KLDiv).

*Pearson’s Correlation Coefficient* interprets both the predicted and the ground-truth saliency maps as random variables, and measures the linear relationship between them as follows:

2 $$\begin{aligned} {\text {CC}}(P,Q) = \frac{\sigma (P,Q)}{\sigma (P) \times \sigma (Q)} \end{aligned}$$

where *P* and *Q* are the predicted and the ground-truth maps, respectively, and ${\text {CC}}(P,Q) \in [-1, 1]$, where positive values indicate positive correlation, values under zero indicate negative correlation, and close-to-zero value indicate no correlation.

*Mean Squared Error* (MSE) measures the point-wise error between the predicted and the ground-truth saliency map by means of a square L2 norm:

3 $$\begin{aligned} {\text {MSE}}(P,Q) = \frac{1}{N}\sum _{i}^{N}(Q(i) - P(i))^2 \end{aligned}$$

where *P* and *Q* are the predicted and the ground-truth maps, respectively, and *i* is the i-th vertex $v_i = (x_i,y_i,z_i)$. The closer the MSE value is to zero, the more similar *P* and *Q* are.

*Kullback-Leibler Divergence* (KLDiv) measures the difference between two probability distributions. We also use this metric as loss function (Sect. 4.3), and its formulation can be seen in Eq. (1).

### Results and comparisons with previous work

Figure 6 shows our model’s predictions for our test set, seen from three different viewpoints each. Our model is able to yield accurate predictions, focusing on the relevant parts of the meshes (e.g., animals’ heads, or some specific regions from the statues), while mostly ignoring the less relevant parts. Interestingly, our model slightly focuses on some other regions (such as the armadillo’s hands) that are less observed in the ground truth, yet are still conspicuous. Besides this qualitative evaluation, we have also resorted to the metrics introduced above to quantitatively measure our model’s performance. The first row in Table 2 shows the results of such evaluation.

**Fig. 7:**
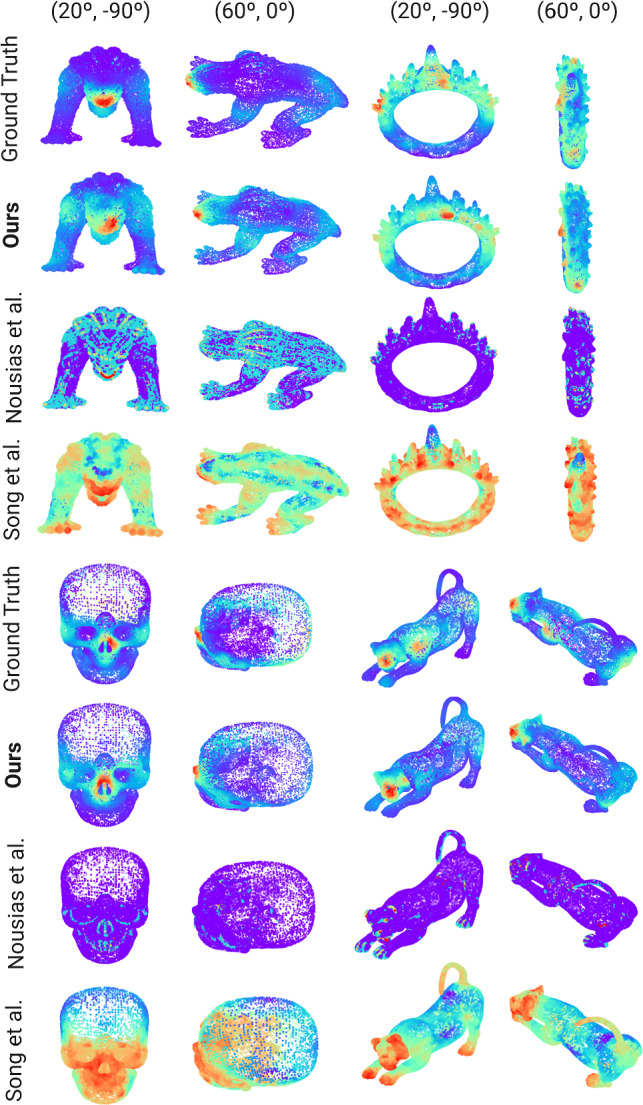
Qualitative results of our comparisons. For each mesh, we show two different viewpoints (parameterized as (elevation, azimuth)) for the ground truth (top row), our model’s result (second row), and the results for the works of Nousias et al. [14] (third row) and Song et al. [15] (fourth row). As in previous figures, color codes saliency. Our model is able to yield predictions much closer to the human ground truth. Nousias et al.’s model ten to focus on very small regions with high frequencies, with very sparse results. SOng et al’s, on the other hand, tends to correctly find the salient regions of the mesh, albeit yielding too high values in too large areas. Quantitative results from this comparison can be found in Table 2, and further discussion can be found in Sect. 5.2

We have also compared our results to the two works that have attempted saliency prediction in 3D meshes before whose code was publicly available, namely Song et al. [15] and Nousias et al. [14]. We have run their model with their default parameters on our test set to obtain their saliency maps. Figure 7 shows, for four different meshes from our test set, the ground truth, our model’s prediction, and the predictions yielded by their models. Note that only our model is trained on real, captured viewing data, and thus it better mimics human behavior. Our model is able to predict maps that better resemble the ground-truth ones. Nousias et al.’s model mainly focuses on small regions with geometrical salient features, such as high-frequency details (e.g., the *lion*’s eyes or the *skull*’s eyes border), failing to capture the wider variability in viewing attention. Song et al.’s model, while focusing on actual relevant regions, overestimates saliency. Quantitative evaluations for this comparison can be found in Table 2.

**Table 2:** Quantitative results of our evaluation

	CC $\uparrow$	KLDiv $\downarrow$	MSE $\downarrow$
Ours	**0.6616 (0.0723)**	**0.3051 (0.1559)**	**0.0204 (0.0033)**
Song et al. [15]	0.1249 (0.1401)	0.7034 (0.3296)	0.3220 (0.1140)
Nousias et al. [14]	0.0570 (0.0976)	1.9618 (0.5187)	0.0759 (0.0189)

We compute three different well-known saliency metrics (see Sect. 5.1) to evaluate our model (first row), and compare it to two available state-of-the-art approaches (Song et al. [15] and Nousias et al. [14]). Best results are in boldface, and each metric indicates whether higher or lower is better. Our model consistently outperforms both previous approaches. Qualitative comparisons can be found in Fig. 7

## Conclusions

In this work, we have presented a deep learning-based approach to saliency prediction in 3D meshes. Different to previous approaches, which have been trained either on low-level geometrical features, or with data gathered in laboratory constrained and unnatural conditions, we have trained our model on a dataset of real, captured gaze and head data from an extensive experiment showing 58 different 3D meshes to more than thirty participants. Then, we have built a computational model upon a state-of-the-art point cloud segmentation network, and trained it on our captured data to predict saliency on unseen meshes. Additionally, we have evaluated our model resorting to well-known saliency metrics, and have qualitatively and quantitatively compared it to available state-of-the-art approaches in saliency prediction for 3D meshes, with our model yielding results that better resemble the ground-truth data.

### Limitations and future work

Several exciting future avenues remain open with this work. As with most data-driven methods, gathering larger, and even more varied—semantically or even geometrically—datasets is key to enhance the model and ensure a more robust generalizability. Besides, while this approach differs from previous ones based on geometric and low-level cues, combining the knowledge from both types of approaches is indeed an natural next step in this problem: Providing our computational model with priors on low-level features could enhance its overall performance. Our viewing data, while less restricted and more natural than previous attempts, has been captured under some particular circumstances: The virtual environment where we showed was empty, and figures were textureless, at a fixed direction, with some fixed light sources, and in uniform, controlled motion. Investigating the effect of semantic context, illumination, or distance, among others, remain an interesting avenue. Moreover, 3D environments are generally designed for users to interact with them; however, in our experiment, participants were seated looking at the shapes. Further studying how attention varies as users interact with the object remains an exciting line of research. Our dataset currently contains viewing data from 32 different participants; recruiting additional participants from wider backgrounds to extend our dataset could further improve the generalizability of our results.

We believe our work is a timely effort toward better understanding attention and 3D, and we will make our code and data publicly available to foster future research.

## Data Availability

Our data, code, and model are available at https://graphics.unizar.es/projects/SAL3D.

## Footnotes

1 http://visionair.ge.imati.cnr.it/ontologies/shapes/

2 http://tosca.cs.technion.ac.il/book/resources_data.html

3 http://watertight.ge.imati.cnr.it/

4 https://www.cc.gatech.edu/projects/large_models/

5 https://free3d.com

6 https://www.turbosquid.com

7 https://www.cgtrader.com

8 https://pupil-labs.com/
